# Supplementary material for: Causal Relationship between Aging and Anorexia Nervosa: A White-Matter-Microstructure-Mediated Mendelian Randomization Analysis
Source: Biomedicines. 2024 Aug 16;12(8):1874. doi: 10.3390/biomedicines12081874 (PMC11351342; doi:10.3390/biomedicines12081874)
Supplement: Supplementary file 1 [file biomedicines-12-01874-s001.zip › Supplementary Figure/Figure S4/Singe SNP plot of MR analysis for TL to ubm-b-1922.pdf]

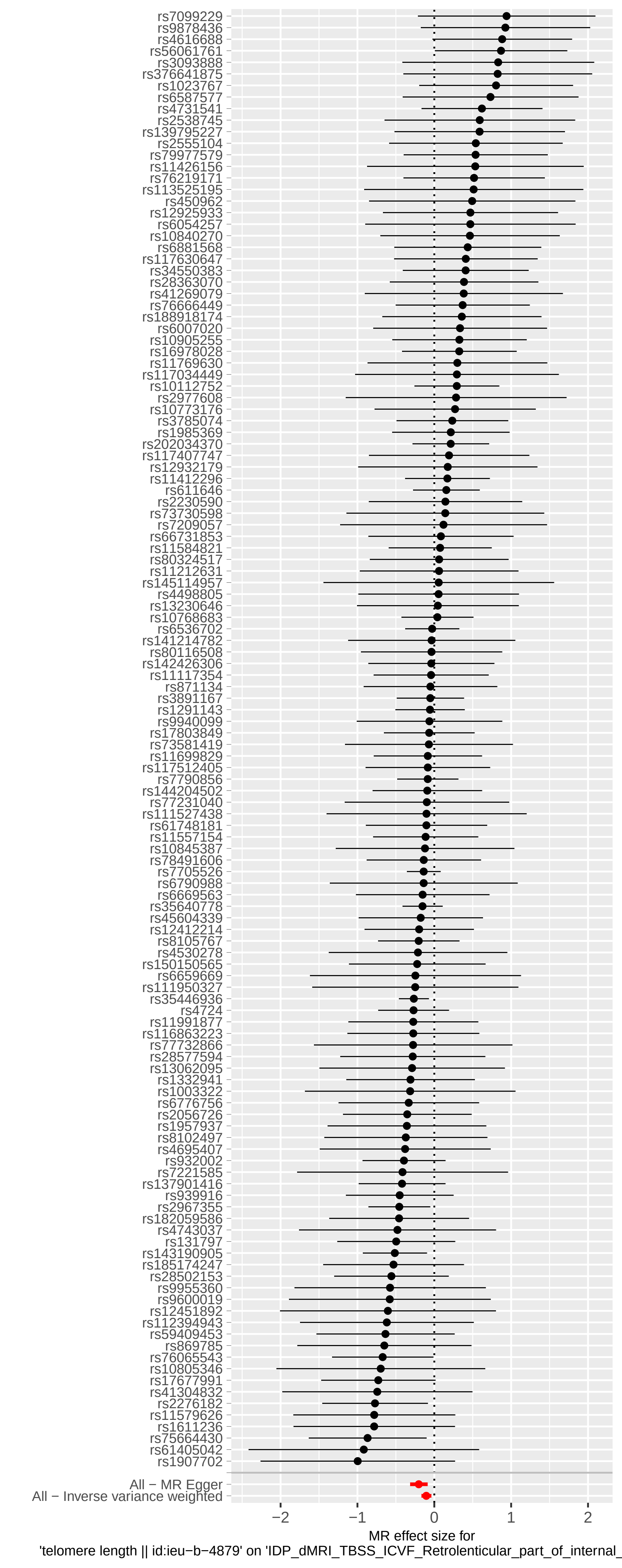
rs7099229
rs9878436
rs4616688
rs56061761
rs3093888
rs376641875
rs1023767
rs6587577
rs4731541
rs2538745
rs139795227
rs2555104
rs79977579
rs11426156
rs76219171
rs113525195
rs450962
rs12925933
rs6054257
rs10840270
rs6881568
rs117630647
rs34550383
rs28363070
rs41269079
rs76666449
rs188918174
rs6007020
rs10905255
rs16978028
rs11769630
rs117034449
rs10112752
rs2977608
rs10773176
rs3785074
rs1985369
rs202034370
rs117407747
rs12932179
rs11412296
rs611646
rs2230590
rs73730598
rs7209057
rs66731853
rs11584821
rs80324517
rs11212631
rs145114957
rs4498805
rs13230646
rs10768683
rs6536702
rs141214782
rs80116508
rs142426306
rs11117354
rs871134
rs3891167
rs1291143
rs9940099
rs17803849
rs73581419
rs11699829
rs117512405
rs7790856
rs144204502
rs77231040
rs111527438
rs61748181
rs11557154
rs10845387
rs78491606
rs7705526
rs6790988
rs6669563
rs35640778
rs45604339
rs12412214
rs8105767
rs4530278
rs150150565
rs6659669
rs111950327
rs35446936
rs4724
rs11991877
rs116863223
rs77732866
rs28577594
rs13062095
rs1332941
rs1003322
rs6776756
rs2056726
rs1957937
rs8102497
rs4695407
rs932002
rs7221585
rs137901416
rs939916
rs2967355
rs182059586
rs4743037
rs131797
rs143190905
rs185174247
rs28502153
rs9955360
rs9600019
rs12451892
rs112394943
rs59409453
rs869785
rs76065543
rs10805346
rs17677991
rs41304832
rs2276182
rs11579626
rs1611236
rs75664430
rs61405042
rs1907702
All − MR Egger
All − Inverse variance weighted
−2
−1
0
1
2
MR effect size for
'telomere length || id:ieu−b−4879' on 'IDP_dMRI_TBSS_ICVF_Retrolenticular_part_of_internal_
